# Supplementary material for: Spatial Variation in Stream-Water Bacterial Communities Across Sites Differing in Wild Chinese Giant Salamander Detection Frequency
Source: Biology (Basel). 2026 Sep 18;15(18):1647. doi: 10.3390/biology15181647 (PMC13604818; doi:10.3390/biology15181647)
Supplement: Supplementary file 1 [file biology-15-01647-s001.zip › Data Related to “Spatial Variation in Stream-Water Bacterial Communities across Sites Differing in Wild Chinese Giant Salamander Detection Frequency”/Microbial Data/result/08.FunctionPrediction/picrust2/heatmap/sample/heatmap.sample.level2.cluster.pdf]

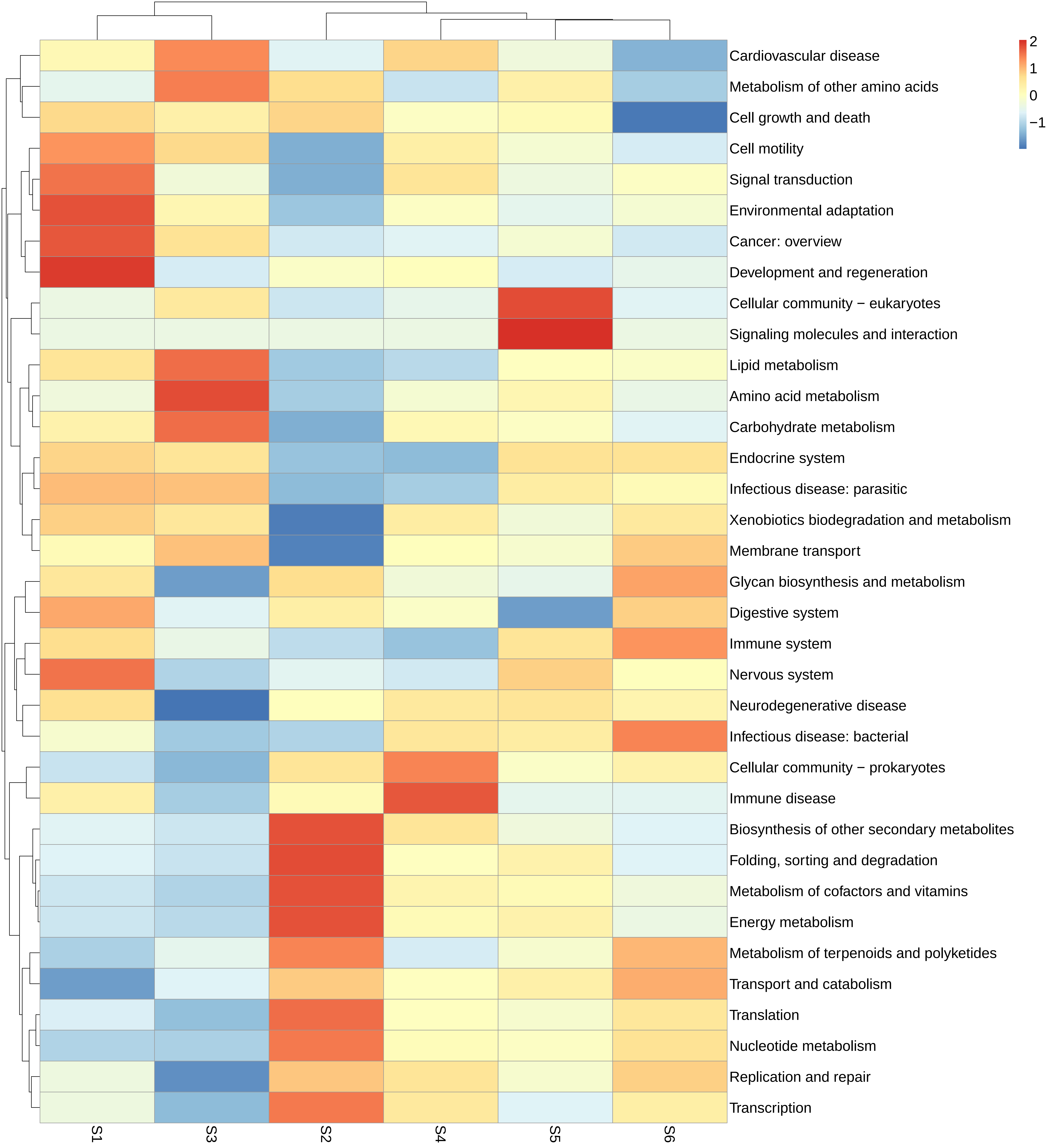
Cardiovascular disease
Metabolism of other amino acids
Cell growth and death
Cell motility
Signal transduction
Environmental adaptation
Cancer: overview
Development and regeneration
Cellular community − eukaryotes
Signaling molecules and interaction
Lipid metabolism
Amino acid metabolism
Carbohydrate metabolism
Endocrine system
Infectious disease: parasitic
Xenobiotics biodegradation and metabolism
Membrane transport
Glycan biosynthesis and metabolism
Digestive system
Immune system
Nervous system
Neurodegenerative disease
Infectious disease: bacterial
Cellular community − prokaryotes
Immune disease
Biosynthesis of other secondary metabolites
Folding, sorting and degradation
Metabolism of cofactors and vitamins
Energy metabolism
Metabolism of terpenoids and polyketides
Transport and catabolism
Translation
Nucleotide metabolism
Replication and repair
Transcription
S1
S3
S2
S4
S5
S6
2
1
0
−1
